# RE-IMAGINING DEMENTIA FAMILY CAREGIVER ENGAGEMENT FOR POST–ACUTE CARE TRANSITIONS: LESSONS LEARNED FROM COVID-19

**DOI:** 10.1093/geroni/igad104.2656

**Published:** 2023-12-21

**Authors:** Ruth Tadesse, Karen Lyons, Glenise McKenzie, Sara Hart, Lee Ellington, Kristin Cloyes

**Affiliations:** Oregon Health & Science University, Portland, Oregon, United States; Boston College, Boston, Massachusetts, United States; Oregon Health & Science University, Portland, Oregon, United States; University of Utah, Salt Lake City, Utah, United States; University of Utah, Salt Lake City, Utah, United States; Oregon Health & Science University, Portland, Oregon, United States

## Abstract

Family caregivers of persons with dementia (PwD) experience three-times more post-acute care transitions (PACT) to skilled nursing facilities than other caregivers. During Covid-19, visitation policies drastically changed, limiting family caregiver engagement in the skilled nursing facility. This was a widespread experience; yet, few studies have examined dementia family caregivers’ perceptions of PACT engagement during Covid-19 restrictions. This qualitative study focused on the findings and lessons learned about the meaning of engagement, barriers, facilitators, and support needs during Covid-19 using an interpretive descriptive approach. Participants (n=15) were mostly female (n=13), adult children (n=8), and the majority (n=9) had a PACT experience during the Covid-19 pandemic. Family caregivers reported improved engagement with the PwD and healthcare team in the acute care setting, because hospital policies on Covid-19 allowed one visitor which gave family caregivers the option to be physically present and advocate for the PwD. However, after the PACT, only essential workers were allowed at the skilled nursing facility making it impossible for family caregivers to visit the PwD and communicate with professional caregivers. Lack of perceived support negatively affected family caregivers’ ability to communicate, share knowledge, advocate, and participate in critical decision making. These Covid-19 challenges highlight the importance of being physically present as a necessary component to engagement which was a form of communication compensation that improves outcomes for the PwD. Future practice policies should view family caregivers as essential to the healthcare team and develop innovative solutions to connect family caregivers to the PwD during Covid-19 or future pandemics.

